# Effects of different types of core training on pain and functional status in patients with chronic nonspecific low back pain: a systematic review and meta-analysis

**DOI:** 10.3389/fphys.2025.1672010

**Published:** 2025-10-16

**Authors:** Xu Bin Guo, Qiuxi Lan, Junyi Ding, Lu Tang, Mingliang Yang

**Affiliations:** ^1^ College of Aviation Physical Education, Civil Aviation Flight University of China, Guanghan, China; ^2^ School of Physical Education, Yulin University, Yulin, China; ^3^ Institute of Aviation Sports, Civil Aviation Flight University of China, Guanghan, China; ^4^ Integrated sports medicine innovation hub for pilots, Civil Aviation Flight University of China, Guanghan, China; ^5^ Department of Aerospace Medicine, Air Force Military Medical University, Xi’an, China

**Keywords:** low back pain, exercise therapy, exercise movement techniques, resistance training, core stability, Systematic review

## Abstract

**Background:**

Chronic non-specific low back pain (CNSLBP) represents a leading cause of global disability, with core training emerging as a promising non-pharmacological intervention. However, the comparative effectiveness of different core training modalities remains unclear. This systematic review and meta-analysis aimed to comprehensively compare the differential effects of three core training approaches—Pilates training, core stability training, and core resistance training—on pain intensity, functional status, and quality of life in adults with CNSLBP.

**Methods:**

A comprehensive systematic search was conducted across four electronic databases (Web of Science, PubMed, Cochrane Library, and Scopus) from inception to May 2025, following PRISMA guidelines. We included randomized controlled trials comparing Pilates training, core stability training, or core resistance training with control conditions in adults aged 18–65 years with CNSLBP ≥12 weeks. Primary outcomes included pain intensity (assessed using Visual Analog Scale [VAS] or Numerical Rating Scale [NRS]) and functional disability (measured by Oswestry Disability Index [ODI] or Roland-Morris Disability Questionnaire [RMDQ]). Secondary outcomes encompassed quality of life measures (SF-36). Risk of bias was assessed using the Cochrane RoB 2.0 tool. Random-effects meta-analysis was performed, with standardized mean differences (SMD) calculated for effect sizes. Meta-regression analysis was conducted to identify optimal training parameters.

**Results:**

A total of 57 randomized controlled trials involving 7,705 participants were included. All three core training modalities demonstrated significant improvements in pain relief compared to controls (SMD = 0.70; 95% CI: 0.58–0.82; p < 0.00001; I^2^ = 47%). Subgroup analysis revealed differential effects: Pilates training showed optimal pain relief effects (SMD = 0.75; 95% CI: 0.58–0.92), followed by core resistance training (SMD = 0.68; 95% CI: 0.56–0.80) and core stability training (SMD = 0.53; 95% CI: 0.34–0.73). For functional status improvement, core resistance training demonstrated the most significant and stable effects (SMD = 0.76; 95% CI: 0.55–0.97; I^2^ = 0%), while Pilates training (SMD = 0.71; 95% CI: 0.13–1.56) and core stability training (SMD = 0.52; 95% CI: 0.33–0.70; I^2^ = 0%) showed moderate improvements. Although the effect sizes for Pilates, core stability training, and core resistance training showed numerical differences, the statistical comparison did not reach significance (P = 0.24) for improving pain and functional status. Meta-regression analysis identified optimal training parameters: core resistance training 3-4 sessions per week (30–45 min per session), Pilates training 2-3 sessions per week (50 min per session, 8–12 weeks duration), and core stability training 3-4 sessions per week (40–60 min per session, 6–8 weeks duration). Training frequency emerged as the strongest predictor of pain improvement in core resistance training (β = 0.48; p = 0.007). All three modalities showed limited effects on mental health components of quality of life.

**Conclusion:**

This study provides the first comprehensive evidence-based comparison of core training modalities for CNSLBP management. Pilates training demonstrates superior effectiveness for pain relief, while core resistance training shows optimal benefits for functional improvement. The identification of specific dose-response relationships and optimal training parameters offers precise clinical guidance for individualized exercise prescription. Core training represents a safe, effective, evidence-based non-pharmacological treatment approach, with clinical application requiring tailored selection based on patient-specific symptoms and treatment objectives. Future research should focus on long-term efficacy evaluation and development of personalized intervention protocols.

**Systematic Review Registration:**

PROSPERO CRD420251054431.

## 1 Introduction

Low back pain has emerged as one of the leading causes of disability worldwide, with epidemiological data showing a 54% increase in disability-adjusted life years from 1990 to 2015. The latest Global Burden of Disease Study reveals that approximately 619 million people globally suffer from low back pain, projected to reach 810 million by 2050 (Hartvigsen et al., 2018; Ferreira et al., 2023). Beyond severely compromising quality of life (Li et al., 2025), low back pain imposes substantial economic burden, with annual healthcare expenditures approaching $100 billion in the United States alone (Katz, 2006). Notably, 80%–90% of cases are classified as non-specific low back pain, lacking clearly identifiable pathological foundations (Behera et al., 2023).

The pathophysiology of chronic non-specific low back pain (CNSLBP) involves complex interactions among structural, biomechanical, neurophysiological, and psychosocial factors (Nijs et al., 2021). Neuromuscular dysfunction, particularly aberrant deep core muscle function, represents a key contributing factor (Cozacov et al., 2022). Research demonstrates significant alterations in transversus abdominis and multifidus activation patterns, with patients shifting from anticipatory to reactive recruitment patterns, severely impacting spinal stability and load transfer mechanisms (Silfies et al., 2009; Zhang et al., 2024). These compensatory alterations persist after pain resolution, necessitating targeted interventions.

Current CNSLBP treatment strategies include pharmacological interventions, physical therapy, and exercise therapy (Savigny et al., 2009). However, medications frequently cause adverse effects, while passive therapies show limited effectiveness for chronic symptoms (French et al., 2004; Peebles et al., 2022). Consequently, exercise therapy has gained prominence as the preferred non-pharmacological intervention, receiving recommendations from major clinical guidelines (Qaseem et al., 2020; Airaksinen et al., 2006). Among exercise interventions, core training has garnered particular attention due to its direct targeting of anatomically critical regions. Systematic reviews demonstrate core training’s superior efficacy compared to general aerobic or flexibility exercises (Wahyuni and Kurnia, 2023; Aparecido Magalhães et al., 2024). Core training optimizes spinal stability through enhanced neuromuscular control, endurance, and strength of the lumbar-pelvic-hip complex (Oliver et al., 2010; Queiroz et al., 2010). Despite widespread acceptance, comparative efficacy among different core training methodologies remains debated. Current paradigms encompass three primary modalities: traditional core stability training based on Panjabi’s spinal stability model emphasizing deep muscle activation (Panjabi, 1992; Xu et al., 2024); Pilates training utilizing six core principles for movement control optimization (Endleman and Critchley, 2008); and general strength training focusing on progressive resistance.

However, evidence regarding relative efficacy presents contradictory findings. While network meta-analyses suggest Pilates superiority (Fernández-Rodríguez et al., 2022), direct comparative studies indicate strength training advantages in pain relief and functional improvement (Miyamoto et al., 2018; Wahyuni and Kurnia, 2023; Alqhtani et al., 2024). More critically, consensus regarding optimal training parameters (frequency, duration, periodization) remains lacking, with significant clinical practice heterogeneity (Seyedhoseinpoor et al., 2022). Although preliminary evidence suggests associations between training parameters and efficacy (Silva et al., 2020), systematic analysis of specific dose-response relationships is absent.

Therefore, this study aims to compare traditional core stability training, Pilates training, and general strength training effects on disability, functional status, and quality of life in CNSLBP patients through comprehensive systematic review and meta-analysis. We seek to elucidate relative therapeutic efficacy and explore dose-response relationships to establish optimal intervention strategies, providing an evidence-based framework for CNSLBP core training prescription development.

## 2 Methods

### 2.1 Literature search strategy

This systematic review and meta-analysis was conducted in strict accordance with the Preferred Reporting Items for Systematic Reviews and Meta-Analyses (PRISMA) statement (Hutton et al., 2015) and the Cochrane Collaboration methodological guidelines (Higgins et al., 2021). The study protocol was prospectively registered with the PROSPERO International Prospective Register of Systematic Reviews (registration number: CRD420251054431).

We conducted a comprehensive systematic literature search across four authoritative electronic databases: Web of Science, PubMed, Cochrane Library, and Scopus, covering the period from database inception to May 2025. The search strategy employed Boolean logic operators (AND, OR, NOT) to systematically integrate keywords, synonyms, and Medical Subject Headings (MeSH terms), ensuring comprehensive coverage of relevant literature. The search framework encompassed three core search domains, each configured with specific search vocabulary (Table 1), with particular focus on identifying randomized controlled trials (RCTs) that compared different exercise intervention modalities—specifically core resistance training, core strengthening training, and Pilates training—regarding their impact on disability, functional capacity, and quality of life in adult populations, especially the improvement effects on patients with chronic low back pain (CNSLBP). Furthermore, in scientific terminology, “core resistance training” is defined as resistance exercises primarily engaging the trunk musculature, including the abdominal, erector spinae, and gluteal regions.

**TABLE 1:** Key search words and synonyms used for each search field.

Population	Exercise	Pain function	Measurement technique
Adult	Core strength	Low back pain	Visual Analog Scale
Older	Core stabil	Chronic low back pain	Oswestry disability index
	Pilates	CLBP	Roland-Morris Questionnaire
	Resistance training	LBP	Numerical Pain Rating Scale
	Strength training	CNSLBP	36-Item Short Form Health Survey

**FIGURE 1:**
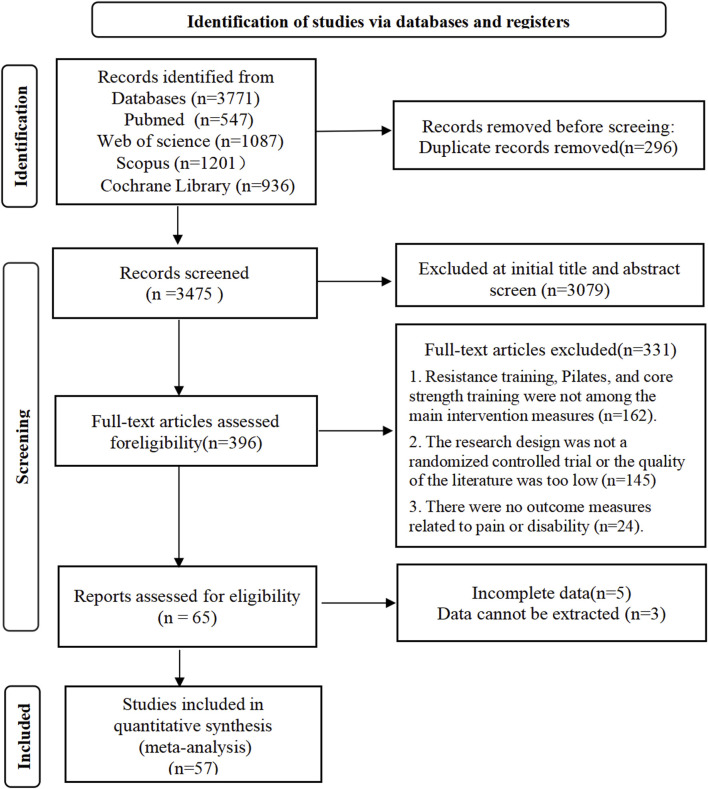
PRISMA flow diagram illustrating the study selection process at different stages.

Prior to data extraction, we established clear exercise category definition criteria, which were validated through peer review procedures to ensure consistency and reproducibility of classification standards. Two independent reviewers (X.B.G. and L.T.) conducted the literature searches separately to minimize selection bias risk. All primary search terms were precisely matched with corresponding MeSH terms and underwent thorough search validation. Additionally, we performed manual screening of reference lists from included studies to identify potentially relevant literature that might have been missed in the initial search. Any disagreements between reviewers were resolved through discussion and consensus, with consultation of a third reviewer when necessary. The PRISMA flow diagram presented in Figure 1 provides a detailed illustration of the systematic search strategy implementation process and clearly indicates the number of studies identified, screened, and ultimately included at each stage of the review.

### 2.2 Inclusion and exclusion criteria

The inclusion criteria for this systematic review were carefully developed based on the PICOS framework (Population, Intervention, Comparison, Outcomes, and Study design) as recommended by the Cochrane Handbook for Systematic Reviews of Interventions (Page et al., 2021). Studies were required to meet all of the following inclusion criteria.

1. Population: Adult patients aged 18–65 years with clinically diagnosed chronic non-specific low back pain (CNSLBP) and symptom duration ≥12 weeks;
2. Intervention: The experimental group received one of the following three exercise intervention modalities: core resistance training, core stability training, or Pilates training, with clearly detailed implementation protocols;
3. Comparison: Control groups included no exercise intervention, standard medical care, or other forms of non-specific physical activity;
4. Outcomes: Studies must have reported at least one primary outcome measure, including pain intensity (measured using standardized tools such as Visual Analog Scale [VAS], Numerical Rating Scale [NRS], or equivalent) or functional disability (measured through Oswestry Disability Index [ODI], Roland-Morris Disability Questionnaire [RMDQ], or other validated assessment instruments). Secondary outcome measures included quality of life-related assessments;
5. Study design: Only randomized controlled trials (RCTs) published in English with accessible full text was included.

Two reviewers (X.B.G. and L.T.) independently performed title and abstract screening using EndNote X9 reference management software, systematically removing duplicate articles and assessing study eligibility based on predetermined criteria. For potentially eligible studies, full texts were retrieved for comprehensive evaluation. Any disagreements between reviewers were resolved through thorough discussion or consultation with a third reviewer (J.Y.D.) to achieve final consensus.

Exclusion criteria included: (1) participants with specific pathological conditions causing low back pain (such as fractures, tumors, infectious diseases, etc.); (2) studies employing combined interventions where exercise effects could not be independently isolated; (3) studies lacking essential data required for meta-analysis despite attempts to contact corresponding authors; (4) non-randomized controlled designs, review articles, case reports, or conference abstracts.

### 2.3 Quality assessment of studies

Methodological quality assessment of included studies was conducted by two independent reviewers (X.B.G. and L.T.) using the Cochrane Risk of Bias tool version 2.0 (ROB 2.0). This tool evaluates potential bias across five core domains: randomization process, deviations from intended interventions, missing outcome data, measurement of the outcome, and selective reporting, with each domain rated as low risk, some concerns, or high risk. Considering the inherent nature of exercise intervention studies where blinding of participants and personnel is difficult to achieve, we focused on examining compensatory measures implemented in each study, including blinding status of outcome assessors, attention control for control groups, and standardization of intervention implementation. Disagreements between reviewers were resolved through discussion, with consultation of a third researcher (J.Y.D.) when necessary. Quality assessment results are presented in visual graphical format.

### 2.4 Data extraction

Data extraction was performed independently by two reviewers (X.B.G. and L.T.) using predefined structured extraction forms. Extracted information included: basic study information (authors, publication year), participant characteristics (sample size, mean age, CNSLBP duration), intervention protocols (exercise type, training intensity, frequency, duration), control conditions, and outcome measurement instruments. Key data extracted focused on changes in pain intensity (measured using Visual Analog Scale [VAS] or Numerical Rating Scale [NRS]) and functional status improvements (assessed through validated tools such as Roland-Morris Disability Questionnaire [RMDQ], Oswestry Disability Index [ODI], and others). In cases of data discrepancies, resolution was achieved through cross-referencing with original texts and, when necessary, contacting original study authors. Complete data extraction results are presented in Supplementary Table S2.

### 2.5 Data synthesis and statistical analysis

Meta-analyses were performed using RevMan 5.4.2 software. Primary outcome measures included pain intensity and functional status, with quality of life as a secondary outcome. Effect size selection was based on measurement tool heterogeneity: weighted mean difference (WMD) was calculated when identical instruments were used, while standardized mean difference (SMD) was employed when different tools were utilized. Heterogeneity was assessed using Cochran’s Q test and I^2^ statistic: I^2^ <25% indicated low heterogeneity, 25%–50% moderate heterogeneity, and >50% high heterogeneity. A fixed-effects model was applied when I^2^ ≤ 50% and P ≥ 0.10, otherwise a random-effects model was used. For studies exhibiting significant heterogeneity (I^2^ >50%, P < 0.05), sensitivity analyses and subgroup analyses (stratified by intervention duration and frequency) were conducted, with meta-regression analysis employed when necessary.

## 3 Results

### 3.1 Study selection

The systematic search across four electronic databases yielded a total of 3,771 articles, comprising 547 from PubMed, 1,087 from Web of Science, 1,201 from Scopus, and 936 from Cochrane Library. After importing into EndNote X9 software and removing 296 duplicate records, 3,475 articles underwent systematic screening. During the title and abstract screening phase, two independent reviewers excluded 3,079 articles that clearly did not meet the inclusion criteria, leaving 396 articles for full-text assessment. The full-text evaluation phase resulted in the exclusion of 331 articles, with primary reasons including: interventions not involving target exercise types (resistance training, core stability training, or Pilates training) in 162 studies, non-randomized controlled trial design or inadequate study quality in 145 studies, and outcome measures unrelated to pain or functional disability in 24 studies. Further assessment of data completeness for the remaining 65 articles led to the additional exclusion of 8 studies due to incomplete data or inability to extract valid data. Ultimately, 57 studies met all inclusion criteria and were included in the quantitative synthesis, comprising 15 core resistance training studies, 21 core stability training studies, 19 Pilates training studies, and 2 studies directly comparing core resistance training with core stability training. The complete literature screening process is illustrated in the PRISMA flowchart shown in Figure 1.

### 3.2 Characteristics of included studies

This systematic review ultimately included 57 randomized controlled trials involving 7,705 patients with CNSLBP, with study sample sizes ranging from 32 to 486 participants and mean participant ages spanning 20.47–69.57 years. The implementation protocols for the three exercise intervention modalities demonstrated certain parameter variations: core resistance training interventions were conducted 2–4 times per week, with session durations of 42–90 min and intervention periods of 8–24 weeks; core stability training was performed 3–4 times per week, with sessions lasting 20–60 min over 4–12 weeks; Pilates training was conducted 2–3 times per week, with session durations of 40–60 min and intervention periods of 2–12 weeks.

Regarding outcome measurements, pain assessment primarily utilized the Visual Analog Scale (VAS, n = 37) and Numerical Rating Scale (NRS, n = 13), with both instruments demonstrating high correlation in pain intensity measurement (r = 0.80–0.95). Functional status assessment was predominantly conducted using the Oswestry Disability Index (ODI, n = 29) and Roland-Morris Disability Questionnaire (RMDQ, n = 16). Quality of life assessment employed the SF-36 Health Survey (n = 8), with primary focus on analysis of the Physical Component Summary (PCS) and Mental Component Summary (MCS) composite scores. Given that VAS (0–100 mm) and NRS (0–10 points) share the same measurement construct and demonstrate high correlation despite different scoring ranges, this study employed linear transformation for data standardization: NRS = (VAS × 10) ÷ 100, to enhance data comparability across different studies and increase the statistical power of meta-analysis. Detailed baseline characteristics of each study are presented in Supplementary Table S2.

### 3.3 Risk of bias in included studies

**FIGURE 2:**
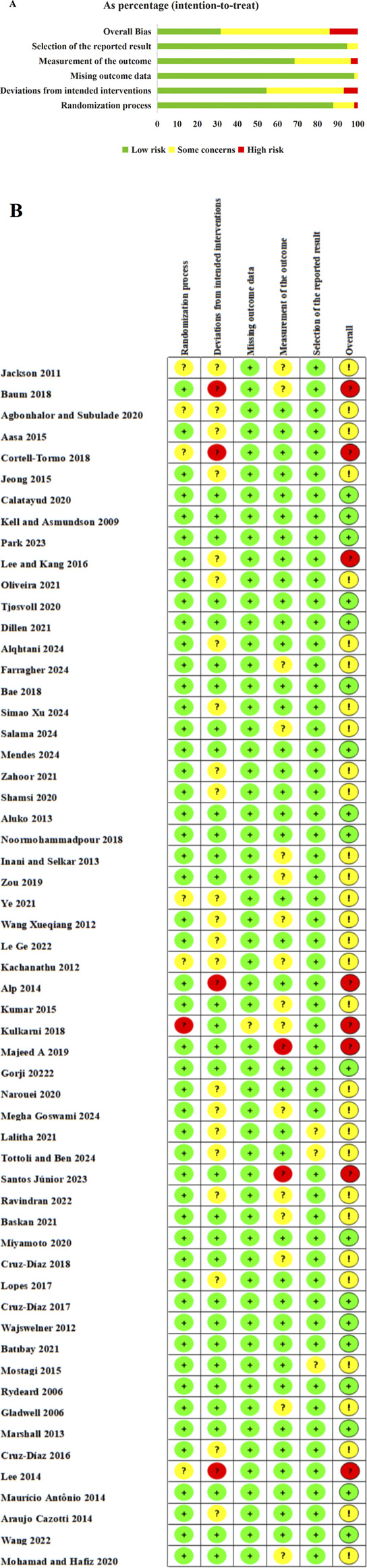
**(A)** Risk of bias graph. **(B)** Risk of bias summary.

All 57 included studies underwent systematic methodological quality assessment by two independent reviewers (X.B.G. and L.T.) using the Cochrane Risk of Bias tool version 2.0 (RoB 2.0), with evaluation encompassing five key domains: randomization process, deviations from intended interventions, missing outcome data, measurement of the outcome, and selective reporting. The overall assessment results revealed that 18 studies (31.6%) were rated as low risk of bias, 31 studies (54.4%) demonstrated some concerns regarding bias risk, and 8 studies (14.0%) were classified as high risk of bias. The comprehensive quality assessment results are presented in Figure 2. Of particular note, considering the inherent characteristics of exercise intervention studies—where complete blinding of participants and intervention providers is difficult to achieve—we specifically examined compensatory methodological measures adopted by each study when assessing implementation bias. These measures included the development and strict supervised execution of standardized exercise protocols, implementation of outcome assessor blinding, adoption of objective measurement indicators to reduce subjective bias, and whether control groups received appropriate placebo interventions or routine care to balance attention effects. Figure 2A illustrates the risk distribution patterns across different bias domains for each study, while Figure 2B presents the summary results of overall bias risk assessment.

### 3.4 Effects of different types of core training on pain (VAS/NRS)

#### 3.4.1 Overall effects

**FIGURE 3:**
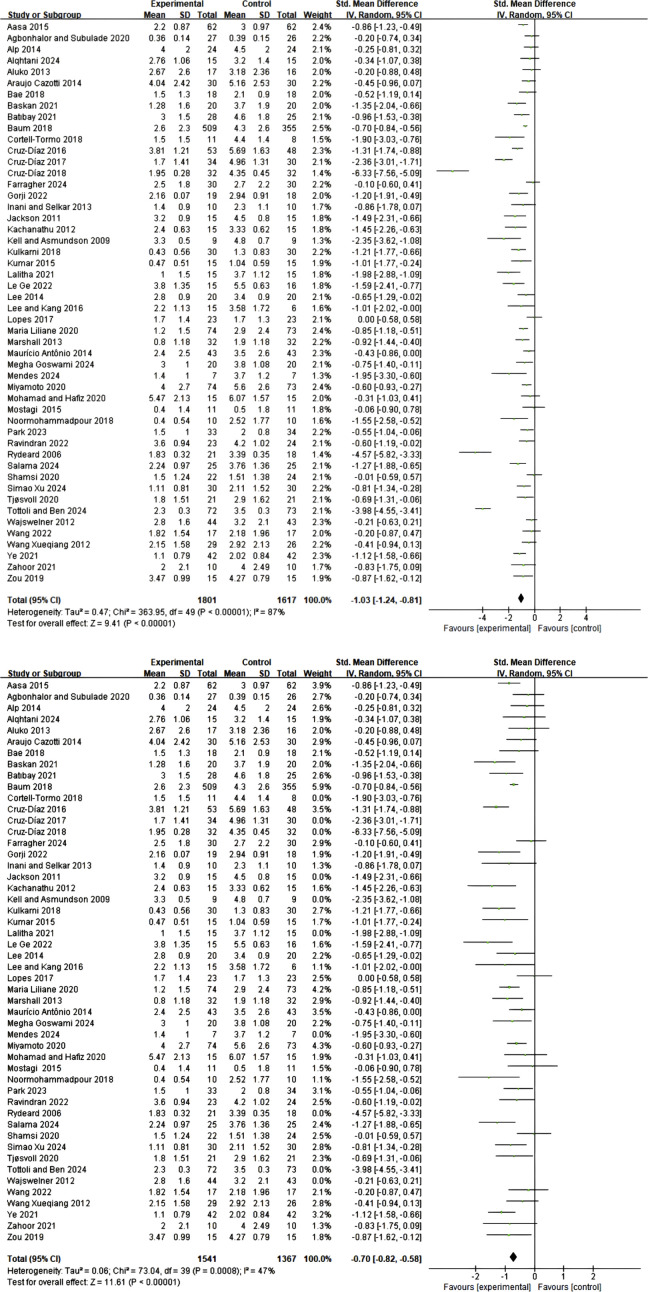
**(A)** Meta-analysis of the effect of exercise on pain scale in CNSLBP patients. **(B)** Meta-analysis of the effect of exercise on pain scale in CNSLBP patients.

A total of 30 studies employed Numerical Rating Scale (NRS) and Visual Analog Scale (VAS) to assess pain intensity. Comprehensive data analysis revealed that, under a random-effects model, Pilates training, core stability training, and core resistance training demonstrated significant advantages over control groups in short-term pain relief (standardized mean difference SMD = 1.03; 95% confidence interval CI = 0.81–1.24; p < 0.00001; I^2^ = 87%), as shown in Figure 3A. Due to substantial heterogeneity between studies, sensitivity analysis was conducted by sequentially excluding 10 potentially outlying studies (Rydeard et al., 2006; Kell and Asmundson, 2009; Jackson et al., 2011; Wajswelner et al., 2012; Cruz-Díaz et al., 2017; Cruz-Díaz et al., 2018; Cortell-Tormo et al., 2018; Lalitha et al., 2021; Mendes et al., 2024; Tottoli and Ben, 2024). Following this exclusion, heterogeneity was significantly reduced (I^2^ = 47%), while the pooled effect size remained statistically significant (SMD = 0.70; 95% CI = 0.58–0.82; p < 0.00001), confirming the robustness of the results (Figure 3B).

#### 3.4.2 Comparative effects of different types of core training

**FIGURE 4:**
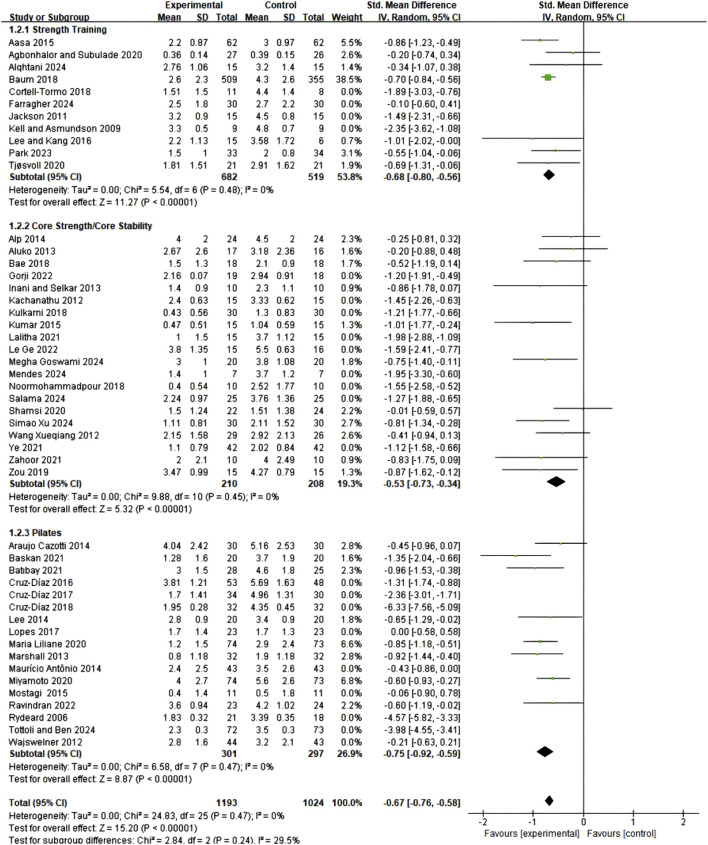
Meta-analysis of the effects of different exercise types on pain scales in CNSLBP patients (subgroup analysis).

Subgroup meta-analysis results demonstrated that all three core training modalities produced significant improvements in pain symptoms among CNSLBP patients, though with varying effect sizes. Pilates training exhibited the most favorable intervention effect (SMD = 0.75; 95% CI: 0.58–0.92; I^2^ = 0%), followed by core resistance training (SMD = 0.68; 95% CI: 0.56–0.80; I^2^ = 0%), while core stability training showed a relatively smaller but still significant effect (SMD = 0.53; 95% CI: 0.34–0.73; I^2^ = 0%), as illustrated in Figure 4. Although the three intervention protocols demonstrated numerical differences in effect sizes, between-group statistical comparison did not reach significance (P = 0.24), and no heterogeneity was observed within individual subgroups (I^2^ = 0%), indicating good consistency and reliability of the results. These findings suggest that all three training modalities represent effective pain management strategies, with clinical selection amenable to individualized decision-making based on patient characteristics and preferences.

#### 3.4.3 Dose-response relationship analysis of training parameters

Resistance Training Parameter Analysis: Univariate meta-regression analysis revealed that among the included training parameters, training frequency had a significant impact on pain improvement effects (β = 0.48, 95% CI: 0.13–0.83, p = 0.007). Each additional training session per week increased the effect size by approximately 0.48 units, with this variable explaining 34.2% of between-study heterogeneity (*R* ^2^ = 34.2%). Session duration (β = 0.005, p = 0.628), total training period (β = −0.009, p = 0.635), and total training volume (β = 0.0001, p = 0.594) showed no significant associations. Multivariate meta-regression analysis further confirmed the independent influence of training frequency, which remained significant after controlling for other parameters (β = 0.51, 95% CI: 0.11–0.91, p = 0.013), with the multivariate model explaining 35.3% of between-study heterogeneity. Based on these findings, the recommended optimal core resistance training protocol consists of 3-4 sessions per week, with session duration flexibly adjusted within the 30–90 min range according to individual patient circumstances, and training periods designed on an individualized basis (Table 2).

**TABLE 2:** Meta-regression: Core resistance Training Parameters and Pain Scale Improvements.

Moderator	Studies (k)	Beta (β)	95% CI	p-value	*R* ^2^ (%)
Univariate Meta-Regression					
Training frequency, sessions/week	10	0.48	(0.13, 0.83)	0.007*	34.2
Single session duration, minutes	10	0.005	(-0.015, 0.025)	0.628	0.0
Total training period, weeks	11	−0.009	(-0.046, 0.028)	0.635	0.0
Total training volume, minutes	10	0.0001	(-0.0002, 0.0004)	0.594	0.0
Multivariate Meta-Regression					
Training frequency, sessions/week	10	0.51	(0.11, 0.91)	0.013*	35.3
Single session duration, minutes	10	0.002	(-0.017, 0.021)	0.837	
Total training period, weeks	10	−0.007	(-0.041, 0.027)	0.688	

Meta-regression of Resistance Training Parameters on VAS/NPRS, Improvements (k = studies; β = regression coefficient; R2 = variance explained). *P ≤ 0.05.

Core Stability Training Parameter Analysis: Univariate meta-regression analysis indicated that session duration had the greatest explanatory power for pain improvement (*R* ^2^ = 5.11%), followed by training frequency (*R* ^2^ = 3.74%). Multivariate meta-regression confirmed that training frequency had the largest impact (β = 0.1281), followed by session duration (β = 0.0120), with both showing positive correlations with pain improvement, while training period showed a weak negative correlation (β < 0). Comprehensive analysis results recommend an ideal core stability training protocol of: 45–60 min per session, 3-4 sessions per week, for a total period of 6–8 weeks. Notably, longer session durations may produce slightly superior pain relief effects, while shorter training periods do not diminish intervention effectiveness, with short-term protocols potentially demonstrating even better results in certain circumstances (Table 3).

**TABLE 3:** Meta-regression: Core strength training parameters and pain scale improvements.

Moderator	Studies (k)	Beta (β)	95% CI	p-value	*R* ^2^ (%)
Univariate Meta-Regression					
Training frequency, sessions/week	17	0.1222	(0.027, 0.217)	0.452	3.74
Single session duration, minutes	17	0.0113	(0.002, 0.021)	0.379	5.11
Total training period, weeks	17	−0.0197	(-0.043, 0.004)	0.594	1.89
Total training volume, minutes	17	0.0001	(0.00003, 0.0002)	0.638	1.48
Multivariate Meta-Regression					
Training frequency, sessions/week	17	0.1281	(0.034, 0.222)	0.212	9.54
Single session duration, minutes	17	0.012	(0.002, 0.022)	0.225	9.54
Total training period, weeks	17	−0.0019	(-0.029, 0.025)	0.884	9.54

Meta-regression of Core Strength Training Parameters on VAS/NPRS, Improvements (k = studies; β = regression coefficient; R2 = variance explained).

Pilates Training Parameter Analysis: Both univariate and multivariate regression analyses failed to identify significant linear relationships between temporal parameters (session duration, training frequency, and total period) and pain improvement effect sizes (session duration: β = −0.0044, *R* ^2^ = 0.0013; training frequency: β = 0.0191, *R* ^2^ = 0.0152; training period: β = 0.0095, *R* ^2^ = 0.0003; multivariate model: *R* ^2^ = 0.0294). Further analysis of high effect size studies (effect size >3.0) revealed common characteristics among studies by (Rydeard et al., 2006; Cruz-Díaz et al., 2018; Tottoli and Ben, 2024): average session duration of 53.33 ± 4.71 min, 2-3 sessions per week, total period of 8–12 weeks, all employing standardized structured protocols with professional instructor guidance. This suggests that the quality and professionalization level of Pilates intervention may be more critical than temporal parameters. Based on high effect size study characteristics, the recommended clinical practice protocol consists of 50–60 min per session, 2-3 sessions per week, for a total period of 8–12 weeks (Table 4).

**TABLE 4:** Meta-regression: Pilates training parameters and pain scale improvements.

Moderator	Studies (k)	Beta (β)	95% CI	p-value	*R* ^2^ (%)
Univariate Meta-Regression					
Training frequency, sessions/week	17	0.0191	(-0.0836, 0.1218)	0.7143	1.52
Single session duration, minutes	17	−0.0044	(-0.0112, 0.0024)	0.2045	0.13
Total training period, weeks	17	0.0095	(-0.0018, 0.0208)	0.0970	0.03
Total training volume, minutes	17	−0.0001	(-0.0006, 0.0004)	0.6825	0.16
Multivariate Meta-Regression					
Training frequency, sessions/week	17	0.0469	(-0.0589, 0.1527)	0.3854	2.94
Single session duration, minutes	17	0.0252	(-0.0035, 0.0539)	0.0853	
Total training period, weeks	17	−0.0231	(-0.1089, 0.0627)	0.5982	

Meta-regression of Pilates Training Parameters on VAS/NPRS, Improvements (k = studies; β = regression coefficient; R2 = variance explained).

### 3.5 Effects of different types of core training on functional status (ODI/RMDQ)

#### 3.5.1 Overall effects

**FIGURE 5:**
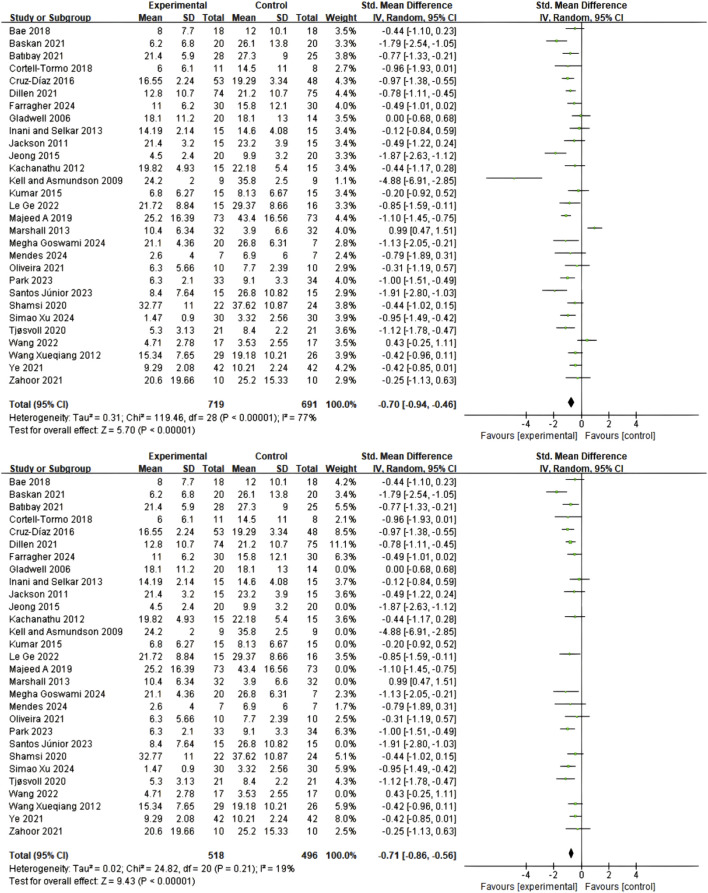
Meta-analysis of the effect of exercise on ODI in CNSLBP patients.

This study employed two widely recognized functional status assessment instruments—the Oswestry Disability Index (ODI) and Roland-Morris Disability Questionnaire (RMDQ)—to evaluate the intervention effects of core training. The ODI assessment included 29 studies with a total of 1,410 participants (719 in experimental groups, 691 in control groups). Meta-analysis results demonstrated that the three types of core training significantly improved participants’ ODI scores (SMD = 0.70; 95% CI = −0.94 to −0.46; P < 0.00001), though significant heterogeneity existed between studies (I^2^ = 76%). Following sensitivity analysis with removal of 8 studies with high heterogeneity (Gladwell et al., 2006; Kell and Asmundson, 2009; Marshall et al., 2013; Jeong et al., 2015; Kumar et al., 2015; Majeed A et al., 2019; Wang, 2022; Santos et al., 2023), heterogeneity was significantly reduced (I^2^ = 19%), while the intervention effect remained stable (SMD = 0.71; 95% CI = 0.56–0.86; P < 0.00001), as detailed in Figure 5.

**FIGURE 6:**
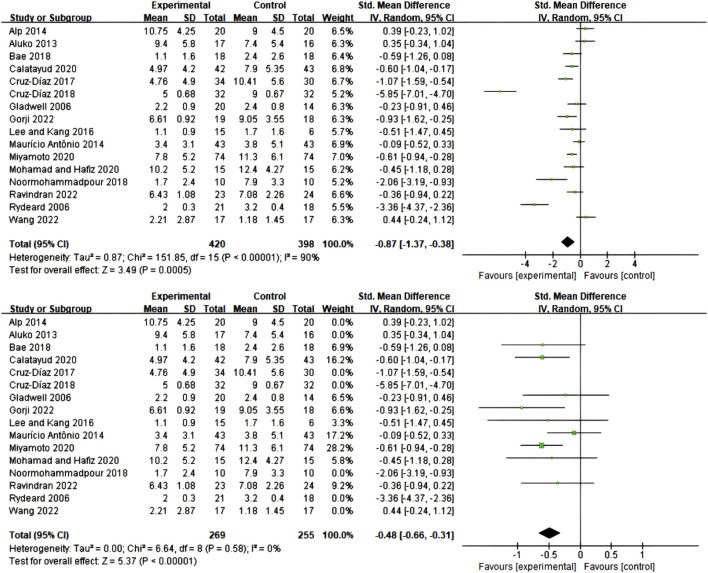
Meta-analysis of the effect of exercise on RMDQ in CNSLBP patients.

The RMDQ assessment included 16 studies with a total of 818 participants (420 in experimental groups, 398 in control groups), similarly demonstrating significant improvement effects (SMD = 0.87; 95% CI = 0.38–1.37; P = 0.0005), though initial analysis revealed high heterogeneity (I^2^ = 90%). Through sensitivity analysis removing 7 studies (Rydeard et al., 2006; Aluko et al., 2013; Alp et al., 2014; Cruz-Díaz et al., 2017; Cruz-Díaz et al., 2018; Noormohammadpour et al., 2018; Wang, 2022), heterogeneity was completely eliminated (I^2^ = 0%), while the intervention effect remained significant (SMD = 0.48; 95% CI = 0.31–0.66; P < 0.00001), as detailed in Figure 6.

Although numerical differences existed in effect sizes between the two assessment instruments, statistical analysis indicated these differences were not significant, demonstrating that regardless of which assessment tool was employed, core training’s improvement effects on functional status were both stable and significant, providing reliable evidence-based support for clinical practice.

#### 3.5.2 Comparative effects of different types of core training

**FIGURE 7:**
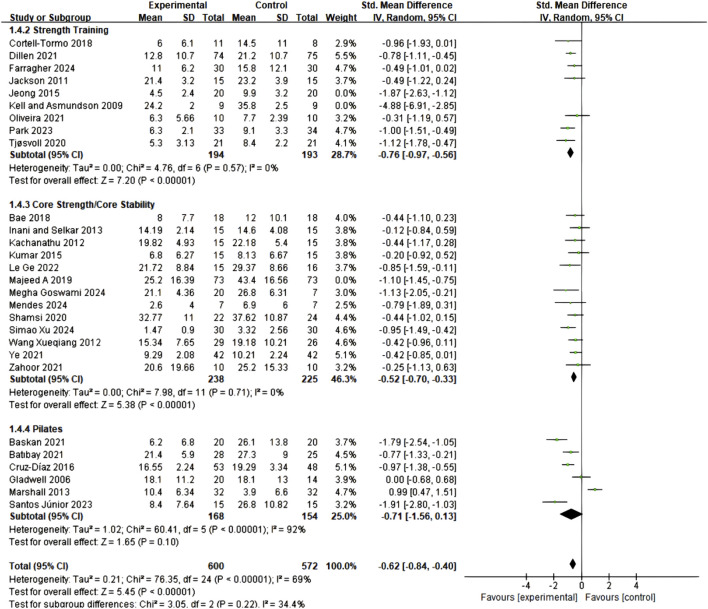
Meta-analysis of the effect of exercise on ODI in CNSLBP patients (subgroup analysis).

Subgroup analysis of 29 studies employing ODI assessment revealed that all three core training modalities significantly improved functional status in CNSLBP patients, though with differences in effect sizes and stability (Figure 7). Core Resistance training demonstrated the most significant and stable therapeutic effects (SMD = 0.76; 95% CI = 0.55–0.97; P < 0.00001), with no heterogeneity between studies (I^2^ = 0%), indicating highly consistent results. Pilates training showed comparable effect size (SMD = 0.71; 95% CI = 0.13–1.56), but this group exhibited high between-study heterogeneity (I^2^ = 92%), which somewhat affected the reliability of results. Core stability training similarly demonstrated positive effects (SMD = 0.52; 95% CI = 0.33–0.70), with relatively low between-study heterogeneity (I^2^ = 0%), yielding relatively stable results. Notably, although numerical differences existed in effect sizes among the three training modalities, between-subgroup comparisons did not reach statistical significance, suggesting that all three training modalities possess clinical therapeutic value. These findings provide important guidance for clinical practice, indicating that the most suitable training protocol can be selected based on individual patient characteristics, treatment preferences, and specific needs, while considering the evidence quality and implementation feasibility of different training approaches.

### 3.6 Effects of different types of core training on quality of life (SF-36)

**FIGURE 8:**
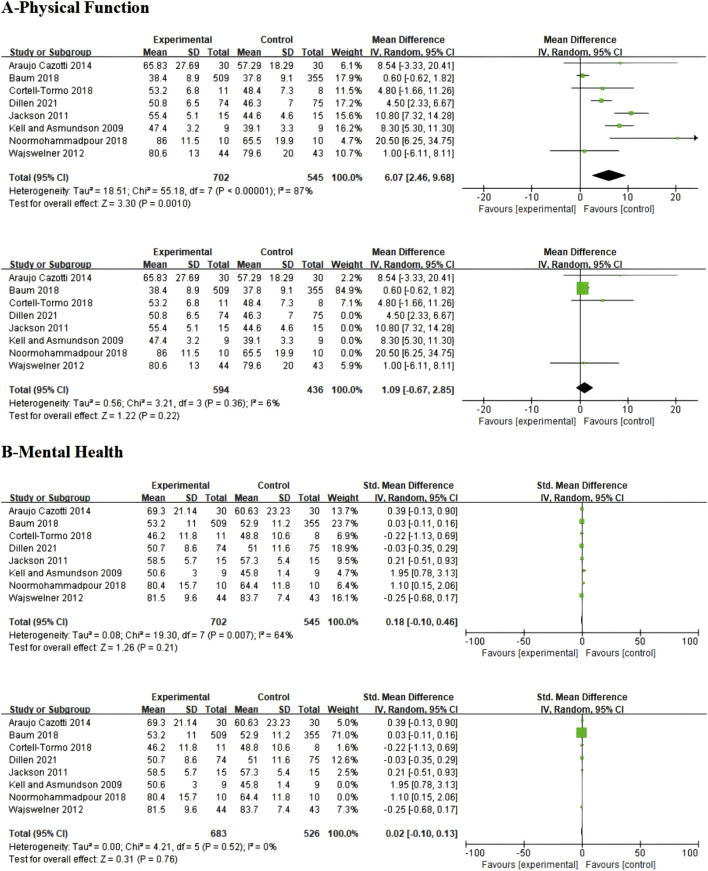
Meta-analysis of the effect of exercise on two dimensions of SF-36 in CNSLBP patients.

For quality of life assessment, this study analyzed two composite indicators from the SF-36 Health Survey: Physical Component Summary (PCS) and Mental Component Summary (MCS). Physical Component Summary analysis included 7 studies, with results showing that core resistance training, core stability training, and Pilates training demonstrated significant effects in improving physical health compared to control groups (SMD = 6.07; 95% CI = 2.46–9.68; P = 0.0010), though extremely high heterogeneity existed between studies (I^2^ = 87%). To reduce heterogeneity effects, sensitivity analysis was conducted excluding 4 high-heterogeneity studies (Kell and Asmundson, 2009; Jackson et al., 2011; Noormohammadpour et al., 2018; Van Dillen et al., 2021), resulting in significantly reduced heterogeneity (I^2^ = 6%). However, the intervention effect was no longer statistically significant (P = 0.22), suggesting that the original significant effect may have been influenced by high-heterogeneity studies, as detailed in Figures 8A.

Mental Component Summary analysis revealed that the three exercise interventions produced only minor improvements in mental health among CNSLBP patients that did not reach statistical significance (SMD = 0.18; 95% CI = −0.10 to 0.46; P = 0.21), with moderate heterogeneity between studies (I^2^ = 64%). After excluding two high-heterogeneity studies (Kell and Asmundson, 2009; Noormohammadpour et al., 2018), heterogeneity reduced to 0%, and the adjusted results further confirmed that the three intervention methods showed no significant differences from control groups in improving mental health (SMD = 0.02; 95% CI = −0.10 to 0.13; P = 0.76; I^2^ = 0%), as detailed in Figures 8B.

These findings suggest that while core training demonstrates significant improvement effects on pain and functional status, its impact on quality of life, particularly mental health, is quite limited. If clinical objectives include improving patient mental health, consideration may need to be given to introducing psychological interventions or other complementary therapeutic strategies.

## 4 Discussion

### 4.1 Summary of main findings

**FIGURE 9:**
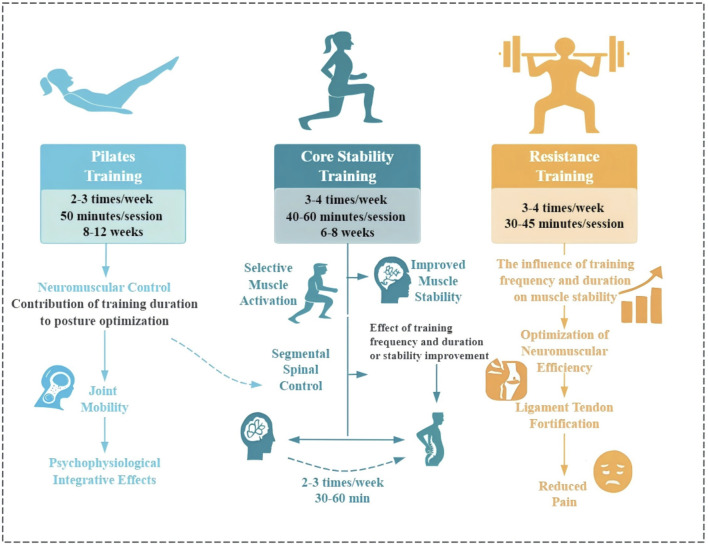
Mechanisms of three core-based therapies in chronic non-specific low back pain relief.

Through systematic review and meta-analysis of 57 randomized controlled trials, this study provides the first comprehensive comparison of the differential effects of three core training modalities (Pilates training, core stability training, and core resistance training) in patients with CNSLBP. The results confirm that all core training interventions significantly improved pain and functional status in CNSLBP patients, with Pilates training demonstrating optimal effects for pain relief (SMD = 0.75) and core resistance training showing the most significant effects for functional status improvement (SMD = 0.76), providing evidence-based support for developing individualized exercise prescriptions. Notably, all three training modalities had limited impact on the mental health dimension of quality of life, emphasizing the importance of adopting a biopsychosocial model in CNSLBP treatment. Figure 9 systematically illustrates the mechanistic pathways through which the three core exercise interventions improve CNSLBP, providing a theoretical foundation for understanding their therapeutic effects.

### 4.2 Differential effects of training modalities

Regarding pain relief effects, subgroup analysis revealed differential outcomes among the three training modalities: Pilates training demonstrated optimal effects, followed by core resistance training and core stability training. These results are consistent with findings from the systematic review by (Yu et al., 2023). The superiority of Pilates training may stem from its multidimensional training characteristics that integrate breathing control, postural awareness, and precise movement control. This approach not only activates deep core musculature but also improves movement control patterns through neuromuscular re-education, potentially triggering multilevel pain modulation mechanisms (Queiroz et al., 2010; Pata et al., 2014; Pat et al., 2014).

Resistance training’s superiority over traditional core stability training in pain relief challenges conventional concepts in clinical practice. This finding supports the perspectives of (Wang, 2022; Mohamad and Hafiz, 2020), suggesting that progressive core resistance training effectively reduces CNSLBP-related pain by enhancing muscle function and spinal load-bearing capacity. The underlying mechanisms may involve muscle hypertrophy, increased tendon strength, and improvements in neural pain inhibitory pathways (Nowotny et al., 2018; Van Dillen et al., 2021).

Regarding functional status improvement, ODI subgroup analysis revealed that core resistance training exhibited the largest and most stable effect, exceeding both Pilates training and core stability training. This finding aligns with research results from (Iversen et al., 2017; Liao et al., 2024), emphasizing the direct association between muscle strength and activities of daily living function. This differential effect supports the viewpoints of (Pata et al., 2014; Carta et al., 2021) that pain relief does not necessarily translate directly into functional recovery.

The advantage of core resistance training in functional improvement is attributed to its direct enhancement effects on muscle strength and endurance, while Pilates training and core stability training focus more on fine motor control and core activation. These latter modalities may produce more significant short-term effects on pain perception but may require longer periods to demonstrate effects in functional restoration (Cruz-Díaz et al., 2018; Wafaa A, 2024).

### 4.3 Impact of training parameters on outcomes

Resistance training parameter analysis revealed that training frequency was the only significant predictor of pain improvement, and this association remained robust after controlling for session duration and total intervention period, consistent with neuromuscular adaptation mechanisms described by (Gomes et al., 2019). Analysis indicated that the optimal intervention frequency was 3-4 sessions per week, with session duration of approximately 45 min. Notably, within the 30–90 min range, session duration showed no significant correlation with effect size, challenging the conventional notion that “longer training yields better results” and supporting the minimum effective dose theory proposed by (Androulakis-Korakakis et al., 2020; Robinson et al., 2024), allowing flexible adjustment of training duration based on patient preferences and feasibility without compromising therapeutic effects.

Pilates training parameter analysis revealed no significant linear relationships between temporal parameters (session duration, frequency, and total period) and pain relief effect sizes, consistent with observations by Silva et al. (2020). Analysis of high effect size studies (>3.0) identified potential optimal practice parameters: average session duration of 50 min, 2-3 sessions per week, intervention period of 8–12 weeks, aligning with research by (Miyamoto et al., 2018). This pattern reflects the unique attributes of Pilates, where therapeutic effects depend more on qualitative factors—including movement precision, breathing coordination, and attention concentration—rather than simple temporal parameters (Volovyk and Pidvalna, 2023), emphasizing the principle that quality supersedes quantity in Pilates training and the critical importance of instructor qualifications (Yang and Chang, 2019).

Core stability training parameter analysis showed that session duration had the most significant impact on pain improvement, followed by training frequency, with multivariate analysis further validating the importance of training frequency. Consistent with findings by (Gottschall et al., 2013; Oliver et al., 2010), the effectiveness of core stability training depends on muscle activation duration and cumulative effects of neuromuscular adaptation. Based on existing literature, 40–60 min training sessions are most effective, with 3-4 sessions per week achieving optimal balance between therapeutic effects and patient adherence. Intriguingly, intervention duration showed a weak negative correlation with pain improvement, possibly reflecting adherence issues in long-term training protocols or training effect plateau phenomena, suggesting that clinicians need to regularly adjust training protocols to maintain patient adherence and sustained therapeutic effects.

### 4.4 Mechanisms of action and mechanistic basis for therapeutic differences among three core training modalities

The differential therapeutic outcomes observed among the three CNSLBP core training modalities stem from their unique intervention mechanisms, and understanding these mechanistic differences provides important theoretical foundations for optimal application strategies in clinical practice (Figure 9).

Pilates training achieves significant pain relief effects through simultaneous action on multiple physiological systems, with intervention mechanisms encompassing three core levels: First, through precise activation of deep core musculature (transversus abdominis and multifidus) to correct delayed muscle activation phenomena characteristic of CNSLBP patients, while incorporating specialized breathing techniques to optimize neuromuscular coordination (Cruz-Díaz et al., 2017; Fontana Carvalho et al., 2020). Second, by enhancing spinal neutral position control capacity to improve load distribution patterns and reduce abnormal spinal stress, with research confirming direct associations between significant increases in muscle thickness and pain reduction (Endleman and Critchley, 2008; Alves et al., 2020). Finally, attention concentration and body awareness principles activate descending pain inhibitory pathways while reducing fear-avoidance behaviors through autonomic nervous system regulation, effectively breaking the pain-tension-anxiety vicious cycle (Barbosa et al., 2015; Parikh and Arora, 2016).

Core stability training is based on Panjabi’s spinal stability system theoretical model (Panjabi, 1992), with specific targeting of local stabilizing muscles (transversus abdominis, ltifidus, pelvic floor muscles) for intervention (Selkow et al., 2017). This training modality imumproves spinal “neutral zone” control capacity, reversing characteristic cortical motor representation changes in CNSLBP patients (Li et al., 2022). Enhanced “muscle cylinder” function optimizes intra-abdominal pressure regulation mechanisms, increases multifidus cross-sectional area, and improves spinal load transfer efficiency (Bernier and Driscoll, 2023). However, its relatively concentrated mechanism of action lacks the progressive loading principles and holistic integration effects possessed by Pilates and core resistance training, which may limit its comprehensive therapeutic effects (Boguszewski et al., 2023).

Resistance training follows progressive overload principles, directly enhancing muscle strength, cross-sectional area, and neural recruitment efficiency (Del Vecchio et al., 2019; Farragher et al., 2024; Guo and Tang, 2024). This intervention modality directly improves functional status by increasing overall lumbar extensor strength and spinal load-bearing capacity (De Oliveira, 2021). Key adaptive changes include: increased type II muscle fiber proportion to correct muscle fiber atrophy commonly seen in CNSLBP patients (Walcott et al., 2011), and stimulation of insulin-like growth factor-1 (IGF-1) to promote tissue repair (Negaresh et al., 2017). Additional benefits encompass pain modulation through endorphin release and inflammatory factor regulation (Chen and Nakagawa, 2023), and enhancement of self-efficacy through quantifiable progress, thereby reducing pain catastrophizing and kinesiophobia (Ryum and Stiles, 2023).

Effect size differences between training modalities directly reflect fundamental differences in their intervention mechanisms. Pilates training’s superior pain relief effects stem from its multi-pathway comprehensive intervention strategy, simultaneously activating multiple descending inhibitory pathways in pain regulation and motor control systems (Fontana Carvalho et al., 2020). While the local stabilization mechanism of core stability training is precise and effective, its overall therapeutic impact may be limited compared to more comprehensive intervention approaches (Boguszewski et al., 2023). Core resistance training’s advantage in functional improvement reflects the direct association between muscle strength enhancement and activities of daily living capacity (Gomes et al., 2019).

The frequency effect of core resistance training is highly consistent with physiological adaptation principles, with 3-4 sessions per week achieving optimal stimulus-recovery balance (Mueller and Niederer, 2020). The non-linear relationship between Pilates training parameters and effect sizes confirms the core principle of “precision over repetition,” emphasizing movement quality over training quantity (Silva et al., 2020). These mechanistic insights provide theoretical support for sequential intervention strategies in clinical practice, such as initial use of Pilates training for pain management followed by core resistance training implementation to optimize function and prevent recurrence (Qaseem et al., 2017).

### 4.5 Strengths and limitations

This study provides evidence-based guidance for CNSLBP clinical management. All three core training modalities are effective but each has distinct advantages: Pilates training is optimal for pain relief, while core resistance training is superior for functional improvement. Clinicians can select appropriate training modalities based on patients’ primary symptoms. Core resistance training: 3-4 sessions per week, 30–45 min per session, with regular protocol adjustments. Pilates training: emphasis on movement quality, recommended 50 min per session, 2-3 sessions per week, intervention period of 8–12 weeks. Core stability training: 3-4 sessions per week, 40–60 min per session, intervention period of 6–8 weeks, avoiding excessive duration that may affect adherence.

In conclusion, our study represents the first large-sample meta-analysis directly comparing three core training modalities (57 studies, 7,705 patients), employing rigorous methodology while simultaneously assessing multiple indicators including pain, function, and quality of life, and providing the first evidence-based evidence for training frequency. However, certain limitations exist: included studies demonstrated heterogeneity in assessment tools and intervention details; most studies had relatively short intervention periods (≤12 weeks), limiting long-term effect evaluation; the three training modalities showed limited improvement in mental health, suggesting the need for comprehensive treatment protocols combining psychological interventions. Variability in terminology used across studies (e.g., “core strength,” “core stability,” “motor control exercises,” “trunk balance exercises”) may have introduced heterogeneity and risks of missing relevant studies despite our broad search strategy. Future research should conduct long-term follow-up trials, develop standardized training protocols, and explore multi-modal combined intervention effects.

## 5 Conclusion

This systematic review and meta-analysis provides the first comprehensive comparative evidence for three core training modalities in CNSLBP management. The results demonstrate that Pilates training exhibits excellence in pain relief, core resistance training shows outstanding effects in functional improvement, while core stability training demonstrates moderate effects in both domains. Meta-regression analysis quantified optimal parameters for each training modality for the first time: core resistance training 3-4 sessions per week (30–45 min per session), Pilates training 2-3 sessions per week (50 min per session, 8–12 weeks duration), and core stability training 3-4 sessions per week (40–60 min per session, 6–8 weeks duration), providing precise evidence-based guidance for clinical practice. The study also identified non-linear dose-response relationships, particularly emphasizing the principle that quality supersedes quantity in Pilates training, which has important implications for clinical practice. Future research should conduct long-term follow-up studies to determine therapeutic durability, develop clinical prediction rules for individualized intervention selection, and explore optimal intervention sequences and combined treatment protocols.

Overall, core training represents a safe, effective, evidence-based non-pharmacological treatment approach for CNSLBP, with clinical application requiring individualized design based on specific patient circumstances and treatment objectives.
