# Supplementary material for: Effects of different types of core training on pain and functional status in patients with chronic nonspecific low back pain: a systematic review and meta-analysis
Source: Front Physiol. 2025 Oct 16;16:1672010. doi: 10.3389/fphys.2025.1672010 (PMC12571568; doi:10.3389/fphys.2025.1672010)
Supplement: Supplementary file 1 [file Table1.docx]

| Supplementary Table 2 (Continued) Characteristics of the included studies. | | | | |
| --- | --- | --- | --- | --- |
| **Study year** | **Mean age**  **(years)** | **Means of**  **intervention** | **Intervention dose**  **(treatment group)** | **Out comes/**  **Major findings** |
| Jackson et al. (2011) | Age: 52 ± 2.7  Study groups:  Intervention (n = 15)  control (n = 15) | Resistance Training | 60 min session, 4 sessions per week for 12 weeks | VAS ODI SF-36  The results suggest that PRT may be effectively applied as rehabilitation for moderately trained recreational athletes with CLBP. |
| Baum et al. (2018) | Age: 54 ± 12.9  Study groups:  Intervention(n = 509)  control (n = 355) | Resistance Training | 42 min session, 2 sessions per week for 8 weeks | VAS SF-36  physical (PQL) and mental quality of life (MQL)  Structured medical training therapies should be recommended as an essential treatment in all stages of non-specific low back pain. |
| Agbonhalor et al. (2020) | Age: ＞18  Study groups:  Intervention (n = 27)  control (n = 26) | Resistance Training | 10 weeks | VAS  It could be recommended that the lumbar muscle strength training program should be considered a key element in the management of patients with LBP and included alongside other conventional physical treatment of LBP among patients with LBP for better outcomes of care. |
| Aasa et al. (2015) | Age: 42 ± 10  Study groups:  Intervention (n =62)  control (n = 65) | Resistance Training | 60 min session, 2 sessions per week for 8 weeks | VAS  The LMC intervention may result in superior outcomes in activity, movement control, and muscle endurance tests compared to HLL, but not in pain intensity and maximal isometric lift strength, in patients with nociceptive mechanical LBP |
| Cortell-Tormo et al. (2018) | Age: 35.6 ± 7.9  Study groups:  Intervention (n =11)  control (n = 8) | Resistance Training | 45-60 min session, 2 sessions per week for 12 weeks | VAS ODI SF-36  Periodized functional resistance training decreased pain and disability and improved HRQOL, balance and physical fitness in females with CLBP, and can thus be used safely in this population. |
| Jeong et al. (2015) | Age: 41.2 ± 5.5  Study groups:  Intervention (n =20)  control (n = 20) | Resistance Training | 50 min session, 3 sessions per week for 6 weeks | ODI  Clinical application of exercise in this study showed that lumbar segmental stabilization exercise plus exercise to strengthen the muscles of the gluteus resulted in a greater decrease in low back pain disability index and increase in lumbar muscle strength and balance ability than lumbar segmental stabilization exercise in chronic low back pain patients receiving the exercise treatments during the same period |
| Calatayud et al. 2020 | Age: 50 ± 12  Study groups:  Intervention (n =42)  control (n = 43) | Resistance Training | 60 min session, 3 sessions per week for 8 weeks | RMDQ  A group-based progressive strength training program can be applied in populations with low back pain with the aim to improve physical function and reduces recurrence and primary care visits more than the most frequently used primary care programs. Importantly, our program can easily be implemented in primary health care at a low cost and with minimum supervision for a group of patients. |
| Kell and Asmundson et al. (2009) | Age: 40.1±8.7  Study groups:  Intervention (n =9)  control (n =9) | Resistance Training | 45-60 min session, 2 sessions per week for 16 weeks | VAS ODI  Physical component summary  Mental component summary  The primary finding was that periodized RT was successful at improving many fitness, pain, disability, and QOL outcome measures, whereas AT was not. This study indicates that whole-body periodized RT can be used by training and conditioning personnel in the rehabilitation of those clients suffering with CLBP. |
| Park et al. (2023) | Age: 44.8 ± 10.8  Study groups:  Intervention (n =33)  control (n = 34) | Resistance Training | 50 min session, 2 sessions per week for 24 weeks | VAS ODI  Although adding abdominal bracing to spinal stability exercise did not affect the changes in the LLA, abdominal bracing improved the spinal extensor strength, pain, and function in patients with CLBP. Therefore, it is recommended to add abdominal bracing to spinal stability exercise to maintain the lordosis angle and to improve CLBP symptoms. |
| Lee and Kang et al. (2016) | Age: 42.7 ± 13.4  Study groups:  Intervention (n =15)  control (n = 6) | Resistance Training | 50 min session, 2 sessions per week for 12 weeks | VAS RMDQ  In conclusion, participating in strength and walking exercises were beneficial to improve lumbar function. Also, the combined exercise program was more effective for reducing pain levels than the strength exercise. |
| Oliveira et al. (2021) | Age: 18-65  Study groups:  Intervention (n =10)  control (n = 10) | Resistance Training | 60 min session, 2 sessions per week for 8 weeks | ODI  The two exercise programs were effective in improving function, flexibility and abdominal strength in patients with chronic, non-specific low back pain. However, there were no statistically significant differences in any of the outcomes in the comparison between groups. |
| Tjøsvoll et al. (2020) | Age: 40 ± 13  Study groups:  Intervention (n =21)  control (n = 21) | Resistance Training | 90 min session, 2 sessions per week for 16 weeks | NRPS ODI  Periodized resistance training with weekly undulating periodization is a feasible training method for this group of individuals with persistent non-specific LBP. A randomized clinical trial should assess the efficacy of such an intervention. |
| Dillen et al. (2021) | Age:42.4 11.8  Study groups:  Intervention (n =21)  control (n = 21) | Resistance Training | 60 min session, 6 sessions per week for 1 weeks | ODI  People with chronic LBP who received MST had greater short-term and long-term improvements in function than those who received SFE. Person-specific MST in functional activities limited owing to LBP should be considered in the treatment of people with chronic LBP. |
| Alqhtani et al. (2024) | Age: 20.47 ± 1.41  Study groups:  Intervention (n =15)  control (n =15) | Resistance Training | 50-65 min session, 3 sessions per week for 6 weeks | VAS  This suggests that the observed differences between the two interventions are not only statistically significant, but also clinically relevant, surpassing the established MCID. |
| Farragh et al. (2024) | Age: 46.5±12.59  Study groups:  Intervention (n =30)  control (n =30) | Resistance Training | 30 min session, 2 sessions per week for 12 weeks | VAS ODI  patients who coMpleted 12 weeks oF resistance training with or without combined neuromuscular control exercises had clinically important reductions in CLBP-related disability, pain intensity, and kinesiophobia. Retraining lumbar extensor neuromuscular control in combination with strengthening exercises yielded no greater improvements in self-reported disability than strengthening exercises alone |
| Bae et al. (2018) | Age: 32.7 ± 6.1  Study groups:  Intervention (n =18)  control (n =18) | Core Strength | 30 min session, 3 sessions per week for 4 weeks | VAS RMDQ ODI  Assisted SUE using new training device can be an effective therapeutic exercise to strengthen dynamic abdominal muscles and improve core muscle activation pattern in NSLBP patients. |
| Simao Xu et al. (2024) | Age: 21.5 ± 0.83  Study groups:  Intervention (n =30)  control (n = 30) | Core Strength | 55 min session, 3 sessions per week for 8 weeks | VAS ODI  Therefore, it can be concluded that core stability training is significantly effective in treating CNLBP in youth, enhancing lower back muscle function. This therapeutic effect is primarily attributed to the improvement in muscle function. |
| Salama et al. (2024) | Age: 26.68 ± 4.72  Study groups:  Intervention (n =25)  control (n = 25) | Core Strength | 40 min session, 3 sessions per week for 12 weeks | VAS  Core stability exercises is a beneficial therapeutic program in reducing pain in patients with chronic non-specific low back pain |
| Mendes et al. (2024) | Age: 27±1  Study groups:  Intervention (n =7)  control (n =7) | Core Strength | 45 min session, 2 sessions per week for 12 weeks | NPRS ODI  The stabilisation exercise programme focused on the core muscles was successful in decreasing the fighter pilots’ chronic lumbar pain. |
| Zahoor et al. (2021) | Age: 20-57  Study groups:  Intervention (n =10)  control (n =10) | Core Strength | 20 min session, 2 sessions per week for 12 weeks | VAS ODI  The study indicates that core stability exercises are more effective than Williams’ flexion exercises for the reduction of non-specific low back pain except for a reduction in pain-related disability. |
| Shamsi et al. (2020) | Age: 38.9±12.2  Study groups:  Intervention (n =22)  control (n =24) | Core Strength | 20 min session, 3 sessions per week for 6 weeks | ODI VAS  The effects of two exercises on pain, disability and antagonist coactivation or imbalance ratios were not different. |
| Aluko et al. (2013) | Age: 35.8±9.1  Study groups:  Intervention (n =22)  control (n =24) | Core Strength | 6 weeks | VAS  The results of this study demonstrated an increase in acceleration accompanied by a reduction in pain, which may suggest that acute nonspecific low back pain may induce the pain-spasm-pain model rather than the pain adaptation model. |
| Noormohammadpour et al. (2018) | Age: 43.3±7.5  Study groups:  Intervention (n =10)  control (n =10) | Core Strength | 8 weeks | VAS RMDQ  This study showed that a multi-step core stability exercise program is a helpful treatment option for improving quality of life and reducing disability and pain in female nurses with CLBP. |
| Inani and Selkar et al. (2013) | Age: 27.8±7.34  Study groups:  Intervention (n =15)  control (n =15) | Core Strength | 30 min session, 3 sessions per week for 4 weeks | VAS ODI  Core stabilization exercises were found to be more effective in reducing pain and improving functional status by decreasing disability of patients with non-specific low back pain in comparison with conventional exercises. |
| Zou et al. (2019) | Age: 58.13 ± 5.38  Study groups:  Intervention (n =15)  control (n =15) | Core Strength | 60 min session, 3 sessions per week for 12 weeks | VAS  Chen-style TCC and CST were found to have protective effects on NF in aging individuals with NLBP, while alleviating non-specific chronic pain. |
| Le Ge et al. (2022) | Age: 64.60 ± 3.71  Study groups:  Intervention (n =15)  control (n =15) | Core Strength | 40 min session, 4 sessions per week for 4 weeks | VAS ODI  Core stability training is an effective intervention for older women with LBP. |
| Ye et al. (2021) | Age: 41.45 ± 10.23  Study groups:  Intervention (n =42)  control (n =42) | Core Strength | 30 min session, 6 sessions per week for 1 weeks | VAS ODI  Suspended core stabilization training has a significant long-term effect in reducing lower back pain and improving waist function in patients with chronic nonspecific low back pain. |
| Wang Xueqiang et al. (2012) | Age: 39.03 ± 4.74  Study groups:  Intervention (n =29)  control (n =26) | Core Strength | 40 min session, 3 sessions per week for 12 weeks | VAS ODI  Our results demonstrate that core stability exercise using unstable, can be more effective decreasing pain, improving trunk muscle endurance and reducing the disability of daily life dysfunction. |
| Kachanathu et al. (2012) | Age: 20.79 ± 2.08  Study groups:  Intervention (n =15)  control (n =15) | Core Strength | 45 min session, 4 sessions per week for 8 weeks | VAS ODI  We conclude that the incorporation of spinal core stabilization exercises in the management of chronic low back pain would have better results than conventional exercises for cases of cLBP in fast bowlers. |
| Alp et al. (2014) | Age: 48  Study groups:  Intervention (n =24)  control (n =24) | Core Strength | 45-60 min session, 3 sessions per week for 6 weeks | VAS RMDQ  Though both of the exercise programs were both found to be effective concerning the areas of pain, endurance, function, and daily living in patients with chronic LBP, the SE group was superior to the HE group in the endurance of dorsal extensors and in the improvement of physical role limitation. |
| Kumar et al. (2015) | Age: 30.47 ± 7.16  Study groups:  Intervention (n =15)  control (n =15) | Core Strength | 40 min session, 3 sessions per week for 6 weeks | NPRS ODI  This study concludes that core muscle strengthening exercise along with lumbar flexibility and gluteus maximus strengthening is an effective rehabilitation technique for all chronic low back pain patients irrespective of duration (less than one year and more than one year) of their pain. |
| Kulkarni et al. (2018) | Age: 20.8 ± 3.04  Study groups:  Intervention (n =30)  control (n =30) | Core Strength | 40 min session, 3 sessions per week for 6 weeks | VAS  The study concluded that statically both the groups had equal effects on reduction of low back pain and improvement of core strength in bharatnatyam dancers. |
| Majeed A et al. (2019) | Age: 39.81 ± 12.58  Study groups:  Intervention (n =73)  control (n =73) | Core Strength | 6 weeks | ODI  The TRICCS protocol is effective in a community-based approach in achieving satisfactory outcomes in CLBP in a period of 6 weeks. Patients with high KSB scores may require cognitive intervention also. |
| Gorji et al. (2022) | Age: 55.16 ± 2.61  Study groups:  Intervention (n =19)  control (n =18) | Core Strength | 45-60 min session, 3 sessions per week for 8 weeks | VAS RMDQ  The treatment with PNE/MCE was more effective in improving pain disability and unipodal static and dynamic balance than treatment with CST. Even so, both treatments were shown to be valid and safe in improving all dependent variables analyzed in women with CLBP. |
| Megha Goswami et al. (2024) | Age: 30-45  Study groups:  Intervention (n =20)  control (n =20) | Core Strength | 2 weeks | VAS ODI  The findings reveal that core stability exercises are more effective than General exercises in chronic low back pain patients. |
| Lalitha et al. (2021) | Age: 30-40  Study groups:  Intervention (n =15)  control (n =15) | Core Strength | 30-45 min session, 4 sessions per week for 8 weeks | VAS  Both the treatment procedures have shown an effective outcome, but core stabilization exercises have proven to be more effective than the general back exercise statistically. |
| Tottoli and Ben et al. (2024) | Age: 35.7 ± 9  Study groups:  Intervention (n =72)  control (n =73) | Pilates | 50 min session, 2 sessions per week for 6 weeks | NPRS  Although Pilates was significantly superior to home exercise for pain and disability, the differences were not considered clinically relevant. However, Pilates did provide significant and clinically relevant differences in utility. |
| Santos Júnior et al. (2023) | Age: 22.47 ± 2.95  Study groups:  Intervention (n =15)  control (n =15) | Pilates | 60 min session, 2 sessions per week for 8 weeks | ODI  Opresente estudo sugere que os exercícios de Pilates podem ser uma escolha para o tratamento de pacientes com dor lombar crônica inespecífica. |
| Ravindran et al. (2022) | Age: 53 ± 12  Study groups:  Intervention (n =15)  control (n =15) | Pilates | 40 min session, 3 sessions per week for 4 weeks | NPRS RMDQ  Pilates Training was found superior to Aerobic Exercise in improving pain and disability among post-menopausal women with non-specific chronic low back pain |
| Baskan et al. (2021) | Age: 41.55 ± 3.39  Study groups:  Intervention (n =20)  control (n =20) | Pilates | 45 min session, 3 sessions per week for 8 weeks | VAS ODI  We recommend using clinical Pilates exercises in clinics instead of the home exercise program for chronic nonspecific low back pain. The recommendation and application of clinical Pilates by physiotherapists will be beneficial in the treatment plan for patients with CNLBP more. |
| Miyamoto et al. (2020) | Age: 47.0 ± 11.5  Study groups:  Intervention (n =74)  Control (n =73) | Pilates | 50 min session, 1-3 sessions per week for 6 weeks | NPRS RMDQ  Adding two sessions of Pilates exercises to advice provided better outcomes in pain and disability than advice alone for patients with NSCLBP; non-specific elements such as greater attention or expectation might be part of this effect. The cost-utility analysis showed that Pilates three times a week was the preferred option. |
| Maria Liliane et al. (2020) | Age: 47.1 ± 14.9  Study groups:  Intervention (n =74)  Control (n =73) | Pilates | 50 min session, 1-3 sessions per week for 6 weeks | NPRS  Different weekly frequencies of Pilates did not accelerate pain improvement in patients with non-specific chronic low back pain. |
| Cruz-Díaz et al. (2018) | Age: 37.9 ± 8.2  Study groups:  Intervention (n =32)  Control (n =32) | Pilates | 50 min session, 2 sessions per week for 12 weeks | VAS RMDQ  Pilates intervention in patients with chronic non-specific low back pain is effective in the management of disability, pain and kinesiophobia. |
| Lopes et al. (2017) | Age: 21.8 ± 3.2  Study groups:  Intervention (n =23)  Control (n =23) | Pilates | 30 min session, 3 sessions per week for 8 weeks | VAS  Pilates exercises immediately improved postural sway and dynamic balance in young adults with non-specific low back pain. |
| Cruz-Díaz et al. (2017) | Age: 37.9 ± 8.2  Study groups:  Intervention (n =34)  Control (n =30) | Pilates | 50 min session, 2 sessions per week for 12 weeks | VAS RMDQ  Equipment based and mat Pilates modalities are both effective in the improvement of TaA activation in patients with CLBP with associate improvement on pain, function and kinesiophobia. Significant differences were observed after 12 weeks of intervention in PMG and PAG with faster improvement in PAG suggesting that, feedback provided by equipment could help in the interiorization of Pilates principles. |
| Wajswelner et al. (2012) | Age: 49.3 ± 14.1  Study groups:  Intervention (n =44)  Control (n =43) | Pilates | 60 min session, 2 sessions per week for 6 weeks | NRPS  An individualized clinical Pilâtes program produced similar beneficial effects on self-reported disability, pain, ftinction and health-related quality of life as a general exercise program in community volunteers with chronic low back pain. |
| Batıbay et al. (2021) | Age: 49.3 ± 10.4  Study groups:  Intervention (n =28)  Control (n =25) | Pilates | 60 min session, 3 sessions per week for 8 weeks | VAS ODI  Both Pilates and home exercises are effective in treating patients with chronic low back pain. UI can be useful for evaluating the core muscle thickness progression of chronic low back pain patients who are undergoing exercise therapy. |
| Mostagi et al. (2015) | Age: 36.1 ± 9  Study groups:  Intervention (n =11)  Control (n =11) | Pilates | 60 min session, 2 sessions per week for 8 weeks | VAS  There were no differences between the Pilates and general exercises with regard to pain and functionality in NSCLBP subjects but general exercises were better than Pilates for increasing functionality and flexibility. |
| Rydeard et al. (2006) | Age: 34 ± 8  Study groups:  Intervention (n =21)  Control (n =18) | Pilates | 60 min session, 3 sessions per week for 4 weeks | NPRS RMDQ  The individuals in the specific-exercise-training group reported a significant decrease in LBP and disability, which was maintained over a 12-month follow-up period. Treatment with modified Pilates-based approach was more efficacious than usual care in a population with chronic, unresolved LBP. |
| Gladwell et al. (2006) | Age: 36.9 ± 8.1  Study groups:  Intervention (n =20)  Control (n =14) | Pilates | 60 min session, 1 sessions per week for 6 weeks | RMDQ ODI  These data suggest that Pilates used as a specific core stability exercise incorporating functional movements can improve non-specific chronic low back pain in an active population compared to no intervention. Additionally, Pilates can improve general health, pain level, sports functioning, flexibility, and proprioception in individuals with chronic low back pain. |
| Marshall et al. (2013) | Age: 36.2 ± 8.2  Study groups:  Intervention (n =32)  Control (n =32) | Pilates | 50-60 min session, 3 sessions per week for 8 weeks | VAS ODI  Inferential statistics suggest greater improvements at 8 weeks, but not 6 months, for the SEG. Inspection of clinically meaningful changes based on a minimum level of adherence suggests no between-group differences. |
| Cruz-Díaz et al. (2016) | Age: 69.57 ± 2.18  Study groups:  Intervention (n =53)  Control (n =48) | Pilates | 40 min session, 2 sessions per week for 6 weeks | NPRS ODI  The resultsl suggest that using Clinical Pilates in addition to physical therapy provides improved results on painl management and functional status for postmenopausal woman with CLBP and that its benefits still linger after one year. |
| Lee et al. (2014) | Age: 34.0 ± 3.3  Study groups:  Intervention (n =20)  Control (n =20) | Pilates | 50 min session, 3 sessions per week for 8 weeks | VAS  PME showed greater improvement in pain level and balance compared with PAE in this research. Since the subjects of this study were patients with low back pain, PME is assumed to have been more suitable and effective because it uses body weight to strengthen core muscles rather than heavier apparatuses as in PAE. |
| Maurício Antônio et al. (2014) | Age: 43.5 ± 8.6  Study groups:  Intervention (n =43)  Control (n =43) | Pilates | 60 min session, 2 sessions per week for 6 weeks | NRPS RMDQ  Equipment-based Pilates was superior to mat Pilates in the 6-month follow-up for the outcomes of disability and kinesiophobia. These benefits were not observed for pain intensity and global perceived effect in patients with chronic nonspecific low back pain. |
| Araujo Cazotti et al. (2014) | Age: 47.79 ± 11.47  Study groups:  Intervention (n =30)  Control (n =30) | Pilates | 50 min session, 2 sessions per week for 13 weeks | VAS RMDQ SF-36  The pilates method can be used by patients with LBP to improve pain, function and aspects related to quality of life (functional capacity, pain and vitality). Moreover, this method has no harmful effects on such patients. |
| Wang et al. (2022) | Age: 26  Study groups:  Intervention (n =17)  Control (n =17) | Resistance Training vs Core Strength | 30 min session, 3 sessions per week for 8 weeks | VAS ODI RMDQ  In the short term, PPCE provides positive effects similar to those of core stability exercise in patients with CLBP. The effective mechanism of PPCE might be the consequence of neuromuscular plasticity and adaptation adjustments. PPCE enriches the choices of treatment for CLBP. Clinical Trial Regist |
| (Mohamad and Hafiz et al. (2020) | Age: 33.57 ± 5.28  Study groups:  Intervention (n =15)  Control (n =15) | Resistance Training vs Core Strength | 2 sessions per week for 6 weeks | RMDQ  In conclusion, both progressive DMST and conventional MGB3 core stability exercise programs are effective for nonspecific chronic LBP rehabilitation. |

.
